# Adult Onset Still's Disease: A Retrospective, Single-Center Study

**DOI:** 10.7759/cureus.10008

**Published:** 2020-08-25

**Authors:** Syed Adeel Hassan, Ali S Choudhry, Somia Jamal, Fahad N Sheikh, Umar Farooque

**Affiliations:** 1 Internal Medicine, Dow University of Health Sciences, Karachi, PAK; 2 Internal Medicine, Lahore Medical and Dental College, Lahore, PAK; 3 Internal Medicine, Abbasi Shaheed Hospital, Karachi, PAK; 4 Pathology, University of Illinois at Chicago, Chicago, USA; 5 Neurology, Dow University of Health Sciences, Karachi, PAK

**Keywords:** adult onset stills disease, aosd, clinical features, laboratory findings, diagnosis

## Abstract

Background

Adult-onset Still's disease (AOSD) is characterized by a classical triad of spiking fever, arthritis, and evanescent rash. It is one of the main causes of hospital admissions for fever of unknown origin and has an extended mean time to diagnosis. Therefore, it remains underdiagnosed relative to its aforementioned time frame. In this study, we attempt to highlight clinical and lab findings associated with AOSD. We then compare our diagnostic results with similar previous studies. Our results should help physicians not to miss this rare entity and make the diagnosis in a reduced time frame.

Materials and methods

This a retrospective, single-center study conducted at Dow University Hospital in Karachi, Pakistan. Thirty patients were enrolled in this study for six months (July 2019-December 2019). All patients were identified and recruited in the medical outpatient department using Yamaguchi's criteria. Written consent was obtained from all patients to access all their clinical charts. Demographics and prior results of laboratory investigations were retrieved from the chart files.

Results

In our study, the mean age of patients was 26.6 years. More specifically, males accounted for 53.3% (n=16) of cases, and females accounted for 46.6% (n=14) of total cases. The most common presenting clinical features included arthralgia (100%, n=30), fever (100%, n=30), myalgia (96.6%, n=29), large joint arthritis (66.6%, n=20), sore throat (50%, n=15), hepatomegaly (40%, n=12), splenomegaly (23.3%, n=7), skin rash (36.6%, n=11) and pericarditis (20%, n=6). Furthermore, none of our patients had cervical lymphadenopathy. The most commonly reported laboratory findings include leukocytosis (100%, n=30), hyperferritinemia (90%, n=27), elevated erythrocyte sedimentation rate (100%, n=30) and abnormal liver function tests (76.6%, n=23). Chest x-rays performed in all patients revealed no abnormalities in 83% of patients (n=25). All patients in our study sample (n=30) tested negative for antinuclear antibodies and rheumatoid factor. It was also noted that the mean duration of diagnosis in our patients was 5.98 weeks. The mean hospitalization period in our patients was 12.5 days. Furthermore, 16.7% of patients (n=5) suffered from disease relapse.

Conclusion

Our study ascertains that the presence of certain clinical and laboratory findings strongly indicate a diagnosis of adult-onset Still's disease.

## Introduction

Adult-onset Still's disease (AOSD) is a multisystemic inflammatory disease of unknown etiology [1]. It is characterized by a triad of spiking fever, arthritis, and salmon-colored evanescent rash [2,3]. AOSD was initially described in 1971 by Bywaters. This clinical entity in adults is thought to be similar to the systemic juvenile idiopathic arthritis (SJIA) in children. It frequently remains underdiagnosed and is one of the main reasons for fever of unknown origin. The majority of patients (75%) are young individuals between the ages of 16 and 35. It has a bimodal age of distribution with two peaks. The first peak affects people within 15-25 years of age. Whereas the second peak affects people within 36-46 years of age. However, several cases with disease onset after the age of 60 have also been reported [4,5]. It tends to predominantly affect females [6]. The incidence of AOSD is estimated at 0.34 cases per 100,000 people [7]. In France, the incidence is estimated to be slightly lower at 0.16 cases per 100,000 people annually [8]. Multiple risk factors have been implicated in the pathogenesis of AOSD. Stress has been suggested as a common risk factor for cases of all ages [9,10]. AOSD is diagnosed using predefined Yamaguchi's criteria or the more recent Fautrel's criteria [11,12].

Since tuberculosis (TB) is an endemic disease in our population, most patients are misdiagnosed as TB and are initiated on antituberculous treatment [13]. Multiple antibiotics are also frequently prescribed before establishing the diagnosis of AOSD. The variability in clinical presentation and lack of specific serologic markers makes the diagnosis of AOSD difficult. It usually necessitates the exclusion of infectious, neoplastic, and autoimmune diseases. Factors that account for a bad prognosis include the presence of pleuritis, interstitial pneumonia, and unremitting fever after a course of prednisolone [14]. Several studies in the literature have been identified which describe AOSD as a diagnostic challenge [15]. Data on specific clinical signs and serological markers for its diagnosis are still insufficient. However, some researchers suggest the role of hyperferritinemia as a diagnostic tool in AOSD [16,17]. In our study, we diagnosed 30 cases of AOSD using Yamaguchi's criteria [18]. Using this study we highlight the striking clinical findings and the possible serological markers that are predominant in our part of the world. We then compare these findings with other studies.

## Materials and methods

This is a retrospective chart-based review in a single-center conducted at a tertiary care hospital in Karachi, Pakistan. We conducted our study at the outpatient and inpatient departments of Dow University Hospital. Our clinical team thoroughly reviewed charts for patients in both inpatient and outpatient clinical settings. Only those patients who met Yamaguchi's criteria were enrolled in the study. A summary of Yamaguchi's criteria is shown in Table 1 [18]. Patients who met the criteria and had a previous history of treated tuberculosis underwent sputum testing and chest x-ray to help justify their inclusion. Furthermore, we excluded patients with active tuberculosis, malignancy, infections, and rheumatic diseases.

**Table 1:** Summary of Yamaguchi's criteria used to screen patients for our study The diagnosis of adult-onset stills disease requires the presence of five criteria with at least two major criteria [18].

Yamaguchi’s Criteria
Major Criteria
Arthralgia >2 weeks
Intermittent fever (>39 degrees Celcius ) for >1 week
Salmon colored rash
White blood cells >10,000 ( >80% granulocytes)
Minor Criteria
Sore throat
Lymphadenopathy
Splenomegaly
Abnormal liver function tests
Negative antinuclear antibodies
Negative rheumatoid factor

Initially, a detailed history and physical examination were conducted for all patients. They then underwent a routine battery of baselines investigations and advanced investigations. Routine investigations included a complete blood count, coagulation profile, liver function tests, erythrocyte sedimentation rate, blood culture, urine culture, and chest x-ray. Advanced investigations included serum ferritin levels, antinuclear antibody levels, rheumatoid factor levels, and autoimmune marker profile.

Data retrieved from case files were entered on to a standard datasheet and analyzed using SPSS 20.0 (Armonk, NY: IBM Corp) and WPS Office (Beijing: Kingsoft). Mean and standard deviation (SD) was calculated for all clinical features of our cohort. The data refers to a cohort of patients recorded between July 2019-December 2019. 

## Results

On careful analysis, 30 patients were diagnosed with AOSD. Of the 30 patients, 14 (46.6%) were females and the remaining 16 (53.3%) were males. The age of the patients ranged from 17-50 years old. Their calculated mean age was 26.66 ± 7.3 years (Table 2). Most patients were from 17 to 30 years old age group (n=26, 86.6%). The remaining cases were above the age of 30 years (n=4, 13.3%). The final tabulation of results from our cohort is presented in Table 2. This table was carefully utilized to assess and derive clinically significant findings. Furthermore, the mean and standard deviation were also calculated for clinically significant variables.

**Table 2:** Tabulation of patient data from our study mg/dL, milligrams per deciliter; mm/hr, millimeters/hour; ug/dL, micrograms per deciliter; IU/l, international units per liter; cu/mm^3, cubic millimeter; ANA, anti-nuclear antibody; RF, rheumatoid factor; ESR, erythrocyte sedimentation rate; SGPT, serum glutamic pyruvic transaminase; WBC, white blood cell

Patients	Gender	Age (years)	Hemoglobin (mg/dl)	ESR (mm/hr)	Serum ferritin (ug/dL)	Elevated SGPT (IU/l)	WBC Count (cu/mm3)	ANA	RF	Chest X-ray	Time to diagnosis (weeks)	Duration of hospitalization (days)
Patient 1	M	17	9.3	99	3260	54	18	Negative	Negative	Normal	2.8	0
Patient 2	F	25	11.2	109	22,567	390	22	Negative	Negative	Normal	3.3	12
Patient 3	F	22	12.1	102	35,871	260	14.12	Negative	Negative	Abnormal	1	20
Patient 4	M	30	10.2	68	952	210	19	Negative	Negative	Normal	10	11
Patient 5	F	26	10	110	8899	96	26	Negative	Negative	Normal	6	21
Patient 6	M	19	9.8	62	2452	144	20.87	Negative	Negative	Normal	7.3	25
Patient 7	F	24	12	105	23.657	150	16	Negative	Negative	Normal	13	10
Patient 8	M	17	11.3	101	17,550	210	18	Negative	Negative	Normal	11	9
Patient 9	F	28	12.4	74	18,320	289	20.1	Negative	Negative	Abnormal	0.2	16
Patient 10	M	30	9.8	88	39,450	360	28	Negative	Negative	Normal	0.5	19
Patient 11	F	20	9.5	63	17,541	320	24	Negative	Negative	Normal	9.2	8
Patient 12	F	27	9.49	91	122	140	16.69	Negative	Negative	Normal	4.6	10
Patient 13	M	29	11.8	60	1,056	250	22	Negative	Negative	Normal	7.8	5
Patient 14	M	25	12.3	79	1,200	366	25.5	Negative	Negative	Normal	10.5	0
Patient 15	F	23	9.4	106	5,690	300	20	Negative	Negative	Normal	4	12
Patient 16	F	19	9.56	67	8,899	310	17.44	Negative	Negative	Normal	12.4	19
Patient 17	M	22	9.83	110	9,200	238	16.56	Negative	Negative	Normal	13	22
Patient 18	F	25	12.2	92	21,500	105	12.84	Negative	Negative	Normal	2	13
Patient 19	M	30	9.9	80	1,542	156	28.51	Negative	Negative	Normal	5	20
Patient 20	M	30	10.4	61	149	382	13.36	Negative	Negative	Abnormal	9	14
Patient 21	M	26	9.68	77	6,409	207	15.59	Negative	Negative	Normal	0	18
Patient 22	F	18	9.3	84	22,654	190	18.47	Negative	Negative	Normal	4	7
Patient 23	M	28	12	82	38,540	240	15	Negative	Negative	Normal	3	13
Patient 24	M	21	11.1	98	26,694	215	12.29	Negative	Negative	Normal	12	8
Patient 25	M	29	9.46	100	29,870	372	24.61	Negative	Negative	Abnormal	1.8	10
Patient 26	F	24	9.38	96	30,045	388	22.41	Negative	Negative	Normal	9	11
Patient 27	F	36	9.91	68	12900	101	23.57	Negative	Negative	Normal	5.3	12
Patient 28	M	39	12.18	106	19,734	99	14.52	Negative	Negative	Normal	6	5
Patient 29	M	50	9.33	60	12,300	136	16.7	Negative	Negative	Normal	1.5	9
Patient 30	F	41	11.67	74	34,345	269	26.69	Negative	Negative	Abnormal	4.4	16
Mean	N/A	26.66666667	10.54966667	85.73333333	14991.15523	231.5666667	19.628	N/A	N/A	N/A	5.986666667	12.5
SD	N/A	7.392136238	1.144029429	17.18044866	12805.6377	100.4715377	4.69509311	N/A	N/A	N/A	4.105062756	6.207614901

All patients in the study presented with fever (>39°) lasting more than a week. Most patients were suffering from fever for the past two to three months at the time of clinical presentation. Varying patterns of fever were duly noted amongst our patients. Most commonly, an intermittent pattern of fever was reported in 83.3% of patients (n=25). The remainder of the patients (16.6%, n=5) experienced continuous fever. A hundred percent of patients presented with arthralgia for more than two weeks (Table 3). The duration of fever and joint pain was identical in most patients. In all patients, arthralgia was confined to the knees and ankles. Polyarticular arthritis was noted in 20 patients (66.6%). A synovial fluid analysis was not conducted in any of these patients. Myalgias were reported in 29 patients (96.6%). However, none of the patients had elevated muscle creatine kinase. The classic evanescent, salmon-colored, maculopapular rash was seen in 11 patients (36.6%). The soreness of the throat was reported in 15 patients (50%). To exclude the possibility of other infections, throat swabs and cultures were conducted. In all 15 (50%) patients, the results of the throat swabs and cultures were negative. Cervical lymphadenopathy was not seen in any patient. On abdominal examination, hepatomegaly was present in 12 (40%) patients. Whereas, splenomegaly was noted in seven (23.3%) patients. These patients underwent an ultrasound to confirm the presence of hepatomegaly and splenomegaly. These findings are depicted in Table 3 below.

**Table 3:** Clinical features of AOSD in our study (), percentage of patients; AOSD, adult onset Still's disease

Clinical Features	Study Results (n=30)
Arthritis	20 (66.6%)
Arthralgia	30 (100%)
Myalgia	29 (96.6%)
Sore throat	15 (50%)
Fever > 39 degrees Celsius	30 (100%)
Skin rash	11 (36.6%)
Hepatomegaly	12 (40%)
Splenomegaly	7 (23.3%)
Lymphadenopathy	0
Anemic	23 (76.6%)
Pericarditis	6 (20%)
Anti-tuberculous therapy	4 (13.3%)
Relapse Rate	5 (16.7%)

On clinical presentation, anemia (Hgb <12g/dl) was noted in 23 patients (76.6%) with mean hemoglobin of 10.54 ± 1.14 g/dl. Elevated white blood cell count was present in all patients (n=30, 100%) with a mean value of 19.628 ± 4.69 cu/mm^3. Abnormal liver function tests were seen in 23 patients (76.6%). The liver enzymes were elevated two to five times the normal values. Of note, serum glutamic pyruvic transaminase was markedly elevated with a mean value of 231.56 ± 100.47 IU/l. Erythrocyte sedimentation rate was elevated in 27 patients (90%) with a mean value of 85.73 ± 17.16 mm/hr. Due to the prevalence of hepatitis in Pakistan, all 27 patients (90%) underwent hepatitis viral serologies to rule out other causes of elevated liver function tests. The results of the viral serologies returned negative for all tested patients. With regards to advanced diagnostics, antinuclear antibodies and rheumatoid factor levels were negative in all patients. Serum ferritin levels were tested in all patients. Surprisingly, serum ferritin levels were elevated in 27 patients (90%) with a mean value of 14991 ± 12805 ug/dL. All patients had undergone a chest x-ray. The results yielded a normal chest x-ray in 25 patients (83%). It was abnormal in five patients (16.66%) who had a past medical history of treated tuberculosis infections. In these patients, healed nodules and fibrotic scars were also seen. These aforementioned findings are derived from Table 2.

The time taken to diagnose the patient is known for all patients in the study. The mean time to diagnosis was reported at 5.98 weeks. The average time that the patient spent in the hospital was 12.5 days. The maximum hospitalization period was 25 days. Furthermore, disease relapse was seen in five patients (16.6%). The minimum relapse was three months. Whereas, the maximum relapse time was noted at two years.

## Discussion

In this study, the majority of the cases were males (53.3%). This is contrary to what has been reported in the literature. Usually, females have been predominantly associated with adult onset Still's disease (AOSD) [7,18]. However, in our study, females accounted for 46.6% of cases. We believe this difference can be due to cultural reasons in our setup. The majority of women do not consider going out to a healthcare center for just a fever. Familial restrictions on females may also contribute to this finding.

The most common initial clinical finding noted in our patients was fever (100%, n=30). Many studies have helped corroborate fever as the most consistent clinical finding. Zeng et al. reported a study where all cases (100%) were reported to have a fever [14]. Owlia et al. conducted a review of over 140 articles, where they also reported fever in more than 99% of cases [21]. Arthralgias were also amongst the most common symptoms found in our patients (100%). Al-Temimi et al. reported that 100% of their cases also presented with complaints of arthralgia [20]. However, Owlia et al. reported that joint symptoms (arthritis/arthralgias) were only found in 64%-100% of the cases they reviewed [21]. Therefore, in our study, the most commonly presenting clinical findings include a spiking fever and arthralgia. Skin rash has been reported in the literature as a very consistent finding in patients with AOSD. The classic rash is a salmon-colored maculopapular eruption. It often accompanies fever spikes and can be seen on the chest, abdomen, and proximal limbs. Al-Temimi et al. reported the presence of skin rash in 80% of cases [20]. Furthermore, Zeng et al. reported that rashes were seen in 88.5% of cases [14]. However, we encountered the presence of rash in only 36.6% of cases in our study. This lower incidence of skin rash can be attributed to the highly pigmented dark skin in our population. As a consequence of which the skin rash wasn’t visible enough. Similar studies from India report only a 12.5% incidence of skin rash in patients with ASOD [21]. This can also be attributed to pigmented skin.

Hepatomegaly was found in 40% of our patients (n=12). Whereas, elevated serum glutamic pyruvic transaminase levels were present in 76 6% of our patients (n=23). Liver dysfunction has been highly associated with AOSD. Andrès et al. reported hepatomegaly (47%) with abnormalities in liver biochemistry in at least 76% cases [22]. Efthimiou et al. reported that 50%-75% of patients have hepatomegaly and liver enzyme abnormalities [23]. Therefore, our findings are similar to the studies conducted by Andres et al. and Efthimiou et al. Liver abnormalities have been suggested secondary to nonsteroidal anti-inflammatory drug use for arthralgia. However, Esdaile et al. proposed that these abnormalities are the true reflection of the underlying disease and not salicylate hepatotoxicity [24]. Pericarditis has been also been implicated in such patients with an incidence of 23.8% [23]. Interestingly, we also report an almost similar incidence rate of pericarditis (20%). Similarly, splenomegaly has also been reported in AOSD patients. Efthimiou et al. reported the presence of splenomegaly in 43.9% of cases [23]. However, we report it in 23.3% of cases in our study.

High ferritin levels have now been associated with AOSD for over two decades [3]. Zeng et al. also reported high ferritin in about 80% of cases in China [14]. Furthermore, Catagay et al. reported that 38% of patients have serum ferritin above 1000 ng/dL [25]. Therefore, all these studies consider a ferritin level >1000 ng/mL as positive for AOSD. On this basis, we also report that 90% of our cases have elevated serum ferritin levels (>1000 ng/mL). This is very significant although the level of elevation may vary among patients. Many other studies have also depicted a positive correlation between AOSD and hyperferritinemia. A study from France assessed the reliability of hyperferritinemia as a diagnostic test for AOSD. It revealed that a fivefold elevation of serum ferritin was 80% sensitive and 41% specific. Similar reports of pretest probabilities have been reported from Japan as well [6]. The usefulness of hyperferritinemia has been restricted because it can be found in other conditions such as sepsis, human immunodeficiency virus infection, leukemia, lymphoma, hemochromatosis, Gaucher’s disease, and hemophagocytic syndrome [26,27].

The mean diagnostic time for patients in our setting was 5.98 weeks. There are multiple proposed factors in our setup that could be responsible for the delay in diagnosis. First, physicians think less of this rare autoimmune disease. As tuberculosis is endemic, patients presenting with a prolonged history of fever are initiated on antituberculous therapy. Another factor is cost associated with the battery of investigations that are required for AOSD diagnosis. Patients are then referred to other medical care facilities or leave against medical advice (LAMA). Apart from poverty, the lack of education adds on for patients to have spiritual misbelieves about their sickness. This causes delays in referral to a physician and eventually their diagnosis.

A total of five patients (16%) relapsed within three to 24 months post-treatment. Interestingly, the relapse rate in our study is significantly lower when compared to other studies. Kim et al. reported a 40% relapse rate in AOSD patients from Korea [27]. Furthermore, another study from China reported a relapse rate of 29.5% [14]. Even though AOSD is a benign condition, relapses prolong the duration of therapy and a likely switch to immunosuppressants. These unfortunate patients exhibit a prolonged period from the beginning of treatment to remission. Thus, it is essential to tailor disease activity at the initiation of treatment with enough corticosteroids. In our tertiary care hospital, there is a huge patient burden. Diagnosing AOSD by its clinical features and lab values will help reduce the use of unnecessary antibiotics and antituberculous therapy on patients. Furthermore, early diagnosis of AOSD is also known to have a good prognostic index.

## Conclusions

Based on our findings, we conclude that certain clinical and laboratory findings highly indicate a diagnosis of AOSD. These include fever, arthralgias, increased liver enzymes, and increase serum ferritin levels. Skin rash, although highly reported in western populations, is inconsistent in our population. Furthermore, arthritis, hepatomegaly, splenomegaly, and pericarditis are regular indicators of AOSD. The consistent elevation of serum ferritin in our study indicates its usefulness as a diagnostic serological marker.
